# Trust, Information and Vaccine Aonfidence in Crisis Settings: A Scoping Review

**DOI:** 10.1002/puh2.70073

**Published:** 2025-06-26

**Authors:** Harriet Dwyer, Luisa Enria, Jennifer Palmer, Shereen Ayub, Nadine Beckmann

**Affiliations:** ^1^ Department of Global Health and Development London School of Hygiene and Tropical Medicine London UK; ^2^ Independent Public Health Consultant Arlington Texas United States

**Keywords:** misinformation, trust, vaccines

## Abstract

**Background:**

In humanitarian crises, reliable and accurate information about health, security and humanitarian aid can be a tool for survival. At the same time, existing social structures and information systems are often disrupted, leading to uncertainty and challenges in interpreting information, including information that may guide individual public health decisions, particularly as part of vaccination programmes. This study aims to systematically explore the existing literature on these dynamics.

**Methods:**

A scoping review was conducted using the key themes: misinformation, infodemic, vaccine confidence and trust with relevant synonyms and subheadings included to build the search strategy. Initial searching was conducted through MEDLINE (Ovid), Embase (Ovid), Global Health (Ovid), PsycINFO (Ovid), Web of Science and SCOPUS, and through hand searching reference lists. Articles were screened and data extracted using Covidence software. A content analysis was used to elucidate common and overlapping themes.

**Findings:**

Forty‐one studies from 14 country contexts as well as 4 from regional and global analyses met the inclusion criteria. The themes identified were (1) the drivers of mistrust, (2) the complexity of misinformation and vaccine confidence and (3) equity and programming with communities.

**Conclusion:**

The scoping review concluded that trust is essential for vaccine confidence in crisis contexts, and intentionally cultivating trust means engaging with historical injustices, politics, power dynamics and information. Vaccine equity, culturally sensitive communication strategies and ensuring interventions are community‐driven should also be central to vaccine programming. Critical knowledge gaps remain about the interplay of trust, information and vaccine confidence in crisis settings and the best strategies that should be adopted to support humanitarian response.

## Graphical abstract

A scoping review systematically exploring the peer‐reviewed literature on trust, information and vaccine confidence in crisis settings. Presenting key themes of mistrust driven from political failings, historical injustices and power imbalances, complexities around misinformation and the need for equity and community driven interventions.

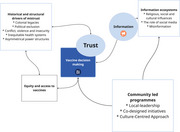

## 1 Background

In protracted conflicts and humanitarian crises, public health information and news about security developments can be tools for survival [1, 2]. Crises, however, can cause the breakdown of social structures vital for community stability and resilience, fuel widespread uncertainty and substantially affect existing information ecosystems—the ways in which people consume, produce, contribute to, interact with and behave around information [2].

Narratives around misinformation and ‘infodemics’ have become a popular way to describe some of the challenges with information ecosystems in crisis settings, including resistance to vaccination and other humanitarian programmes. Misinformation refers to unverified information that does not have secure standards of evidence, often thriving where people are faced with uncertainty and challenging decisions [4]. The World Health Organization (WHO) defines an infodemic as an overabundance of information, both accurate and not [5].

Successful vaccination programmes, on the other hand, rely heavily on access to reliable information and, crucially, trust [6, 7]. Trust itself, however, is often regarded as ambiguous: difficult to articulate, investigate and build within programmes [8]. Often defined as a relationship that exists between individuals, as well as between individuals and a system, it usually involves one party accepting a vulnerable position, assuming the best interests and competence of the other [7]. In crisis contexts, trust is often rendered fragile, impacted by historical injustices, political dynamics and inequitable health systems [9, 10, 11, 12]. These processes have public health implications in crisis contexts where community‐level vaccination programmes are often hampered by reduced trust in authorities and systems alongside an increased risk of vaccine‐preventable disease [13].

The WHO Strategic Advisory Group of Experts (SAGE) on immunisation has defined three key domains of influence driving hesitancy around vaccines: confidence (trust in the safety or efficacy of the vaccine), convenience (ease of access) and complacency (perception of the risk of disease and importance of immunisation) [14]. The first domain, vaccine confidence, implies a level of trust in the vaccine, the vaccinator or other health professional and in those who make the decisions about vaccine provision (the policymaker) [7]. In crisis contexts, the interplay of dynamics around vaccine confidence, information and trust are not extensively evidenced. The aim of this review is to synthesise the existing evidence on this topic, while identifying areas which should be addressed in future research.

## 2 Study Design

The scoping review was conducted according to the JBI (formally the Joanna Briggs Institute) guidance for scoping reviews [15] with methods developed in line with the approach presented by Arksey and O'Malley [16] and provides a broad synthesis of the existing evidence and identification of gaps in perspectives that may be helpful to address [17]. Given the nascency of this research area, the inclusive approach of the scoping review allows for the consideration of various types of evidence, including qualitative, quantitative and mixed‐methods studies, to capture the complexity of these dynamics.

The scoping review protocol was registered in the Open Science Framework: https://osf.io/ne7X4/.

### 2.1 Search Strategy

**FIGURE 1:**
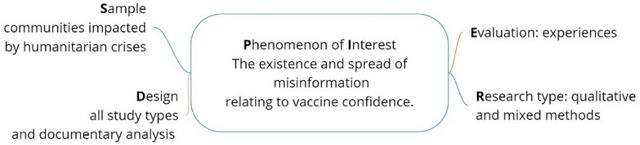
The SPIDER framework [18].

The literature search focused on answering the question, *What is the current evidence about the interplay of trust, information consumption and vaccine confidence in crisis settings?* The SPIDER tool for qualitative evidence synthesis [18] was used to frame the search strategy and identify relevant studies, although quantitative and mixed methods studies were also included (Figure 1). The *sample* was communities impacted by crisis, and the phenomenon of interest was the existence and spread of misinformation relating to vaccine confidence. All study types and documentary analysis were included from the literature (design). The evaluation part of the framework linked to the notion of experiences and the research type was qualitative and mixed methods.

The databases searched were MEDLINE (Ovid), Embase (Ovid), Global Health (Ovid), PsycINFO (Ovid), Web of Science and SCOPUS. Reference lists were also hand searched for any additional relevant works. After piloting the search strategy in all databases, the final search was conducted on 21 June 2024. The search strategy is presented in Table A1.

### 2.2 Eligibility and Screening

Studies were included based off the criteria listed in Table 1. It is important to note that studies were included in contexts where it was deemed that the humanitarian needs were sufficiently large and complex to require significant external assistance and a multi‐sectoral response, with engagement of international humanitarian actors and external resources [3]. Therefore, in addition to conflict settings and acute disasters, disease outbreaks in a fragile context (including COVID‐19) and polio eradication were included.

**TABLE 1:** Inclusion/exclusion criteria.

	Inclusion	Exclusion
*Publication type*	Peer‐reviewed articles and reports Academic theses and dissertations	Non‐English publications Grey literature
*Study design*	Empirical studies, reviews and scoping reviews Qualitative, quantitative and mixed‐methods research Opinion pieces, editorials, commentaries	
*Context/population*	Humanitarian crises, conflict, outbreaks in fragile contexts and related contexts	Stable contexts
*Concepts*	The phenomenon of interest (misinformation and infodemics) Vaccine confidence and vaccine hesitancy Humanitarian/crisis contexts or global studies that have implications for humanitarian response Trust	Other vaccine and humanitarian response related topics
*Timeframe*	No restriction on publication date	

Articles were initially imported into EndNote [19], and duplicates were removed. They were then imported into Covidence [20], missed duplicates were removed, and H.D. conducted the initial title and abstract screening. H.D. and S.A. then conducted a full text review of 99 works in accordance with the inclusion and exclusion criteria included in Table 1. Disagreements in 31 of the articles reviewed were resolved through discussion with reference to the inclusion and exclusion criteria. A total of 41 works met inclusion criteria for data extraction and analysis.

### 2.3 Data Extraction and Synthesis

An extraction template was developed by H.D. in Covidence and piloted with five articles (see Supporting Information section.) This included extraction of general information and data variables, including study characteristics, methods, key themes, key findings and implications for research and practice. H.D. and S.A. conducted independent and blinded data extraction and resolved conflicts in consultation following the completion of the extraction in Covidence.

Frequency counts and a descriptive content analysis were conducted for data relating to study characteristics. Following guidance provided by JBI, a content analysis was conducted manually to synthesise emerging themes and implications for public health practitioners and future research [21, 22]. The results have been reported in line with the Preferred Reporting Items for Systematic reviews and Meta‐Analyses extension for Scoping Reviews (PRISMA‐ScR) Checklist (see Supporting Information section) [23].

## 3 Results

### 3.1 Characteristics

**FIGURE 2:**
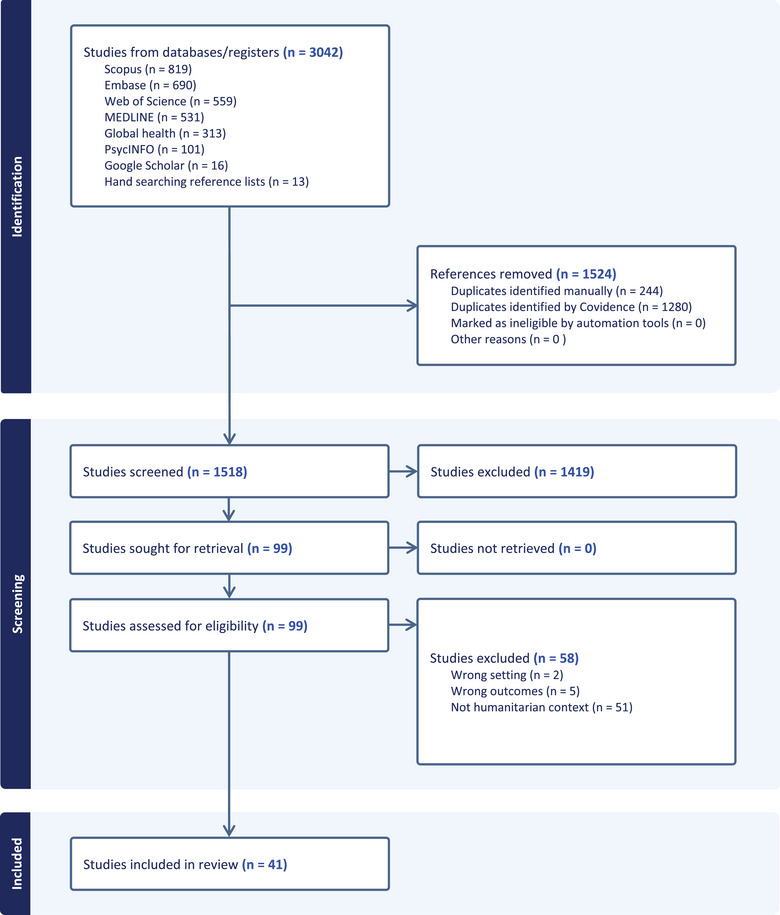
PRISMA flowchart generated via Covidence [20].

**FIGURE 3:**
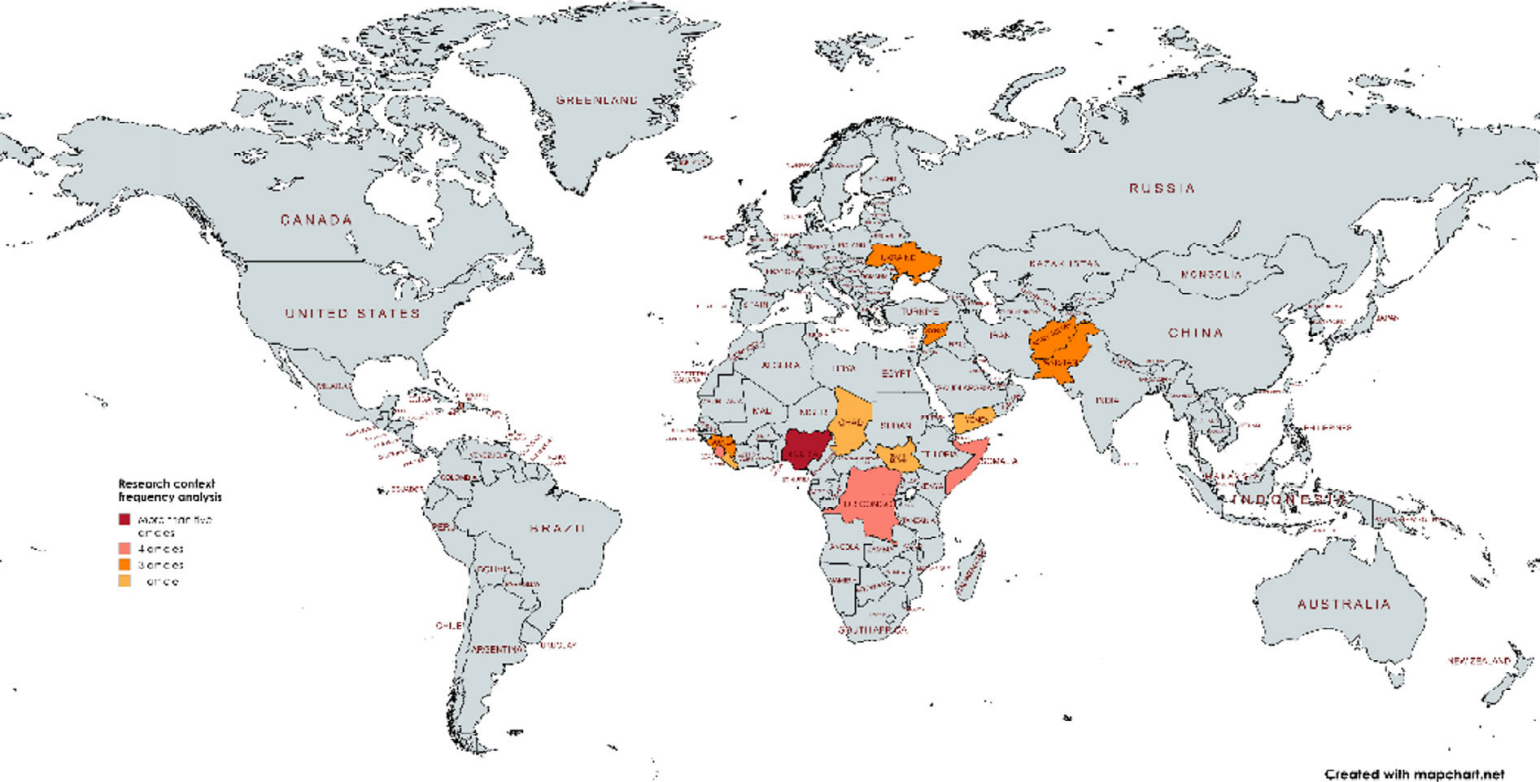
Global overview of geographic distribution of research contexts generated via https://www.mapchart.net/ [24].

Initial database and hand searches yielded 3042 articles. After duplicates were removed, abstract and title screening and full text screening, a total of 41 studies were included for final analysis (detailed in the PRISMA flowchart Figure 2.) The included studies are detailed in a table in the Supporting Information section. A significant number of studies (8) focused on Nigeria. Four studies have been conducted in Haiti, Sierra Leone and the Democratic Republic of Congo (mapped in Figure 3.) The primary settings for the studies were classified within the domains: *epidemics within fragile* contexts (*n* = 14), *COVID‐19 in fragile* contexts (*n* = *16*) and *global analyses on the key* dynamics (*n* = 7) Institutional affiliations of included studies spanned 43 countries (Table 2).

**TABLE 2:** Summary of study characteristics.

Summary of characteristics	
Total number of included studies	41 studies
Study types	Qualitative, cross‐sectional, systematic reviews, commentaries and case studies
Time period of publication	2005–2024
Geographic distribution: research context	Afghanistan, Chad, Democratic Republic of Congo, France, Guinea, Haiti, India, Italy, Nigeria, Pakistan, Philippines, Sierra Leone, Somalia, South Sudan, Syria, Tajikistan, Uganda, Ukraine and Yemen
Geographic distribution: research institution	Afghanistan, Australia, Belgium, Canada, Chad, DR Congo, Democratic Republic of the Congo (DRC), Denmark, France, Germany, Ghana, Guinea, Haiti, India, Jordan, Kenya, Lebanon, Lesotho, Liberia, Malaysia, Mexico, Netherlands, Nigeria, Pakistan, Peru, Philippines, Poland, Qatar, Rwanda, Russia, Sierra Leone, Singapore, Somalia, South Africa, South Sudan, Spain, Sweden, Switzerland, Syria, Tanzania, United Arab Emirates (UAE), United Kingdom (UK), Uganda, Ukraine, United States of America (USA) and Yemen
Crisis types	Epidemics (Ebola, COVID‐19, polio), conflict and protracted crises
Most common data collection methods	Surveys, interviews, focus groups and document analysis

The most common study type was qualitative research (over 35%), followed by cross‐sectional studies (23%). Over 28% of articles were text/commentary/reports. The studies employed a range of methods with interviews (*n* = 14) and document analysis (*n* = 10) being the most prevalent.

### 3.2 Emerging Themes

**FIGURE 4:**
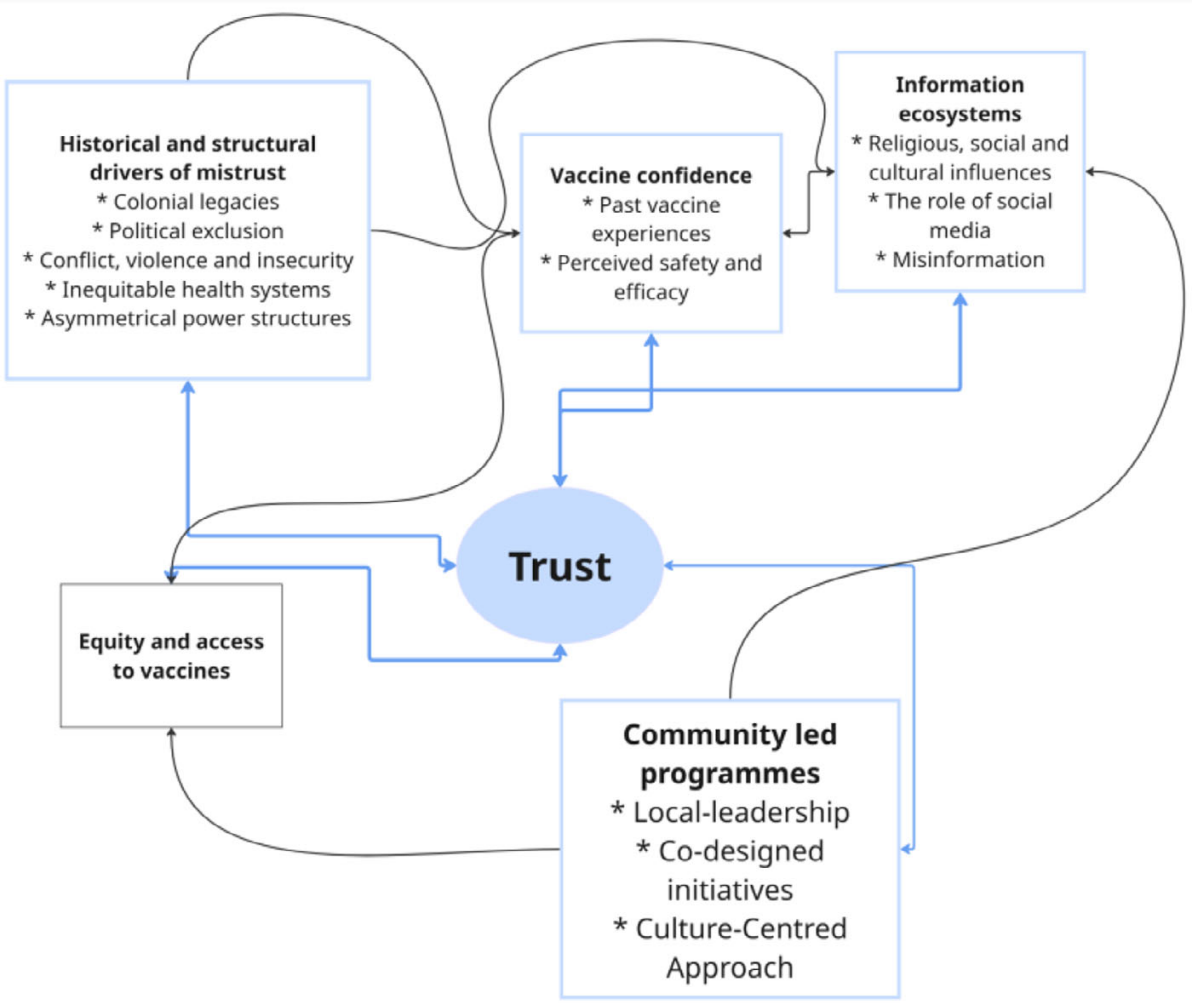
Conceptual map illustrating the relationship between historical, structural and social influences on trust and vaccine confidence in crisis contexts. The diagram highlights how trust is shaped by intersecting factors, including structural barriers, historical injustices, information ecosystems, as well as programmatic emphasis on health system equity and community‐led programmes.

The overarching themes that were identified from the literature are (1) the drivers of mistrust, (2) the complexity of misinformation and vaccine confidence and (3) equity and programming with communities. Although the findings are presented as discrete themes with sub‐themes, they are interconnected and overlap, highlighting the interplay of these dynamics in context (Figure 4).

#### 3.2.1 The Drivers of Mistrust

##### 3.2.1.1 Political Failings Shape Perceptions

Douedari, Enria and Vinck all conclude, through qualitative research and cross‐sectional survey, that low institutional trust, particularly in governments and health systems, and often rooted in negative social and economic experiences, plays a pivotal role in low vaccine confidence [25, 26, 27, 28]. Corruption within health systems and governments, like those documented in Afghanistan and Syria, particularly around vaccine procurement and distribution, was found to exacerbate low institutional trust [29, 30]. In conflict affected areas, fragmented governance structures and inconsistent service delivery contributed to negative perceptions of institutions and the health services they provide [25, 31].

Several studies emphasised how health information, particularly during crises, becomes politicised [31, 32]. In Guinea, for example, debates about the significance of COVID‐19 included assertions that the pandemic was a means of social control or to distract from critical political issues [31]. Different actors, including governments and non‐state actors, may promote narratives to align with their agendas, including through various anti‐vaccination (anti‐vax) movements [26]. This manipulation can create confusion and mistrust, making it challenging for individuals to make informed decisions about vaccination.

##### 3.2.1.2 Power Structures Within the Humanitarian Model

Six qualitative studies articulated the power imbalances in humanitarian contexts between international organisations (providing humanitarian aid), local governments (partners and custodians), civil society and communities [27, 33]. The dynamics often reflected the lack of agency and control that local communities have over the presence and action of international organisations [11]. The dynamics influence trust, confidence in official information and the success of vaccination programmes [11, 34]. The lack of agency and control experienced by local communities and interventions, including vaccination programmes, reinforces feelings of being disregarded, further undermining trust [12].

Perceptions of exploitation where international actors and local elites are seen as profiting from outbreaks and crisis deeply erodes trust. These dynamics were exemplified by the ‘Ebola business’ narrative in the Democratic Republic of Congo, which suggested that international organisations and local elites profited from the Ebola outbreak response [11], and in Nigeria, where suspicion about western biomedicine stems from historical events like the Pfizer Trovan trials, a series of unethical trials conducted by Pfizer in 1996 during a meningitis outbreak, where children were administered the antibiotic Trovan without adequate consent. It has been argued that Pfizer took advantage of a vulnerable situation and did not follow medical protocols [6]. These suspicions resurfaced during polio vaccination campaigns and helped fuel a boycott of the polio vaccine by community and religious leaders [6].

Local power structures, such as patriarchal systems where household decisions are taken by fathers, or systems that challenge notions of fairness, were also seen to shape trust in vaccination campaigns [12]. In Somalia, Abdullahi, for example, identified that social mobilisation efforts focusing on mothers sidelined fathers, excluding them from decision‐making processes related to their children's health [34]. This created friction with health workers, subsequently hindering paternal approval for vaccines and impacting broader community acceptance.

##### 3.2.1.3 Historical Mistrust

Several studies highlighted the profound and enduring impact of historical injustices on vaccination efforts.

Experiences of exclusion, memories of historical oppression, and contemporary experiences of structural violence, underfunding of healthcare, and rising inequality shape attitudes to vaccines [26]

Past experiences of exploitation, unethical medical practices, violence, exclusion and marginalisation create a legacy of mistrust that continues to shape perceptions of vaccines [12, 35, 36, 37]. Colonial powers often used medicine as a tool for control and exploitation, conducting unethical experiments on colonised populations and implementing policies that prioritised the needs of the colonisers over the colonised through the selective provision of healthcare—favouring certain groups or individuals over others [11, 31]. This history has left deep scars and fostered a distrust of Western medicine, which can extend into contemporary vaccination programmes. Ginai, Yahya and Obadare's studies on the polio boycott in Nigeria in 2003 point to the unethical Pfizer Trovan trials as a case of exploitation [6, 36, 37]. These experiences fuel narratives of medical paternalism and reinforce scepticism towards vaccination campaigns, particularly those perceived to be driven by Western interests, including, for example, the testing of new pharmaceuticals in low‐income settings when people who will benefit are in high‐income countries [6].

#### 3.2.2 The Complexity of Misinformation and Vaccine Confidence

##### 3.2.2.1 The Conditions in Which Misinformation Flourishes

Nearly a quarter of studies highlighted misinformation as a barrier to vaccine acceptance, particularly in crisis contexts where access to accurate information may be limited [35, 36, 37, 38, 39, 40, 41, 42]. The sources emphasise that the existence and acceptance of rumour or misinformation should not be dismissed as a matter of an ‘ignorant public’ but a complex issue intertwined with deeper issues of mistrust and marginalisation and heavily influenced by social and cultural contexts and post‐colonial wounds [26, 43, 44].

Rumours, misinformation and alternative expertise that accompany a view of non‐acceptance are also often publicly portrayed as manifestations of ignorance ‐ at best, a lack of information, or, at worst, an inability or unwillingness to engage with scientific fact…. Dismissing misinformation as ignorance, rather than seeing it highlights local concerns, obscures the social commentaries and political critiques that the narratives reveal [33].

Information does not remain static. Local contexts and evolving global events shape how people understand and respond to public health interventions. Several studies refer to ‘embedded meanings’ or social meanings to describe how individuals appropriate, reinterpret and share information within specific social and cultural frameworks [31, 33].

##### 3.2.2.2 Religious and Cultural Influences

Etienne‐Mesubi, Ghinai and Mohamed all detailed in their studies the significance of religious and cultural beliefs in shaping individual interpretations of vaccine information. Religious leaders were seen in particular to influence vaccine acceptance or hesitancy, especially in contexts where religious authority holds significant weight [35, 36, 40]. For example, in northern Nigeria, religious leaders played a pivotal role in shaping public perceptions of the polio vaccine, directly contributing to the vaccine boycott [6, 36, 37]. It was primarily driven by assertions by religious and political leaders that the vaccine was contaminated with anti‐fertility agents and HIV, leading to a resurgence of polio cases in Nigeria and spreading the virus to neighbouring countries [36, 37].

##### 3.2.2.3 Social Media's Role

Ali and Ittefaq's studies on polio eradication programmes in Pakistan highlighted the impact of social media misinformation, citing that the spread of misinformation, particularly through platforms such as Facebook, Twitter and YouTube, is seen by public health responders as a critical challenge [45, 46].

Misinformation about the polio vaccine on social media has led to significant increases in vaccine refusal rates. A viral false rumour in April 2019 caused widespread panic, resulting in mob violence, hospital burnings, and a five‐day suspension of the polio eradication campaign. Over two million children have gone unvaccinated since the incident [46].

False claims about vaccine safety and efficacy have often been intertwined with pre‐existing societal tensions [47]. In Ukraine, for example, disinformation campaigns, used as hybrid war tactics, capitalised on a health system already facing credibility issues against a backdrop of political instability and vaccine supply challenges [48]. Disinformation campaigns leveraged online platforms to fuel scepticism around vaccination, having a lasting effect on vaccine confidence [48].

Several studies highlighted, however, that over‐emphasising the role of social media in relation to the spread and impact of misinformation neglects the broader social, political and historical contexts that shape information consumption [33, 44]. Sources emphasise the importance of understanding local contexts and the processes by which individuals interpret and share information to uncover ‘embedded meanings’ rather than dismiss these instances as merely rumours [31, 33].

#### 3.2.3 Equity and Working With Communities

##### 3.2.3.1 Equity

Several studies emphasised that when discussing vaccine hesitancy, it is crucial to consider both demand and supply‐side factors [11, 32, 33]. Systematic barriers include limited healthcare infrastructure, inadequate resources and challenges in vaccine distribution [38, 39, 47]. Geographic barriers, including mountainous terrain, or lack of transportation and insecurity also hindered access [29, 30, 35]. Abdullahi et al. present these inequities in Puntland, Somalia, where one participant, a mother, in their qualitative study stated:

There are two types of people, those who reside in the towns and those who reside in the countryside. The people who need the vaccination the most are those living in the countryside. But the health workers don't have cars, they have limited time, and they will tell you themselves that they cannot always reach people. They will admit that they don't have the time and transportation that is needed to reach everyone. [34]

Marginalised communities often face additional barriers to vaccination due to factors such as poverty, discrimination and lack of healthcare and information [37, 38, 39, 49, 50]. Sources also illustrate that in some crisis contexts, vaccination (particularly for COVID‐19) is of lower priority than more immediate threats like malnutrition and insecurity caused by conflict [6, 31, 36, 49].

Ensuring that vaccines are easily accessible and affordable, especially in communities with healthcare barriers, is a vital strategy for increasing acceptance [38, 51].

##### 3.2.3.2 Programming With Communities

In 18 studies, community engagement was identified as a key process to build trust and confidence in vaccines and tackle misinformation and infodemics in context. For these efforts to be effective, communities need to be engaged in the design, implementation and evaluation of programmes [10, 26, 37, 38, 52].

Genuine collaboration with community leaders, including religious leaders and local authorities, is reported in conclusions by numerous studies as critical, especially in contexts where they hold significant community influence [11, 35, 36, 37, 53, 54].

Effective community engagement goes beyond simply providing information or addressing misinformation. Studies encourage the establishment of a respectful, culturally contextualised, two‐way dialogue with community members to understand concerns and address questions and to amplify community needs to policy makers [6, 11, 12, 27, 28, 35, 49, 50, 55, 56].

##### 3.2.3.3 Effective Communications Strategies

Transparent communication from public health authorities was identified as a key lever for building trust. Within public health campaigns, it was concluded that to be effective, messaging should be clear and accurate, tailored to specific audiences while taking into account cultural background, literacy levels and existing beliefs [39, 40, 42, 51, 52, 55, 56, 57, 58, 59, 60]. In relation to vaccines this includes clear communication about vaccine development, safety and efficacy, while acknowledging uncertainty and the limitation of public health interventions [55, 59, 60].

Proactive and context‐specific strategies were encouraged when using strategic communications to address misinformation, including monitoring for rumours and misinformation, using trusted communications channels and involving credible sources, such as healthcare workers and community leaders, to debunk false claims [28, 39, 41, 45, 46, 56].

It was recommended that instead of dismissing local beliefs and practices as misconceptions or ignorance, effective communication strategies should strive to understand and integrate these perspectives into health messaging [27, 38, 39]. This approach, it is argued, can enhance trust.

## 4 Discussion

In synthesising the evidence, this review has demonstrated that there is a complex interplay of trust, information and vaccine confidence in crisis contexts. The synthesis of the content has allowed the exploration of common themes and, despite varied methods, studies generally reached similar conclusions around the importance of culturally sensitive and community driven interventions.

### 4.1 Centring Trust

Trust, within the context of humanitarian crises and public health emergencies, cannot be assumed. Trust in civic authorities does not simply exist; rather, it is shaped by the interplay among historical, political, social and cultural factors. Much of the literature highlights the generational impact of historical injustices on population‐wide trust in health systems and health interventions. In these contexts, mistrust is often a rational response to past and present experiences of exploitation and marginalisation [12, 35, 37]. For example, past instances of unethical medical practices which led to the polio vaccine boycott in Nigeria have left a legacy of mistrust in healthcare systems and interventions still present today [26, 61]. This legacy gets passed down through generations, shapeshifts and takes on new meaning in new circumstances of uncertainty, like humanitarian crises [31].

Mistrust is fuelled from the experience and perception of powerlessness [9]. Crisis contexts, particularly protracted and layered ones, are perfect conditions for systems of trust to be altered and scepticism of outside intervention to grow. The sources illustrate how power imbalances between international organisations, national governments and local populations can hinder the effectiveness of humanitarian interventions because of mistrust [6, 10, 11, 34, 55]. As demonstrated during Ebola outbreaks in the Democratic Republic of Congo, for example, top‐down approaches often fail to consider local knowledge, beliefs and practices, further exacerbating mistrust [11].

In response, much of this literature recommends a shift away from external actors within public health programmes. Community leaders, religious leaders and community health workers play a crucial role in localising global health. Key to this is a movement away from solely biomedical approaches in complex contexts towards strategies that listen to community concerns, address long‐standing grievances and intentionally seek to co‐design solutions [6, 27, 49].

This reinforces the idea that trust must be intentionally cultivated, not assumed, particularly in humanitarian settings marked by inequality and historical injustices. The Culture‐Centred Approach (CCA) [62, 63] offers a framework for understanding how trust is fostered when communities are not only included in communication efforts but when their lived experiences, cultural knowledge and structural realities shape the design and delivery of health interventions. More recent work has extended this framework to the importance of building ‘voice infrastructures’: platforms that allow communities to participate meaningfully in health governance and advocate for structural change [64]. These insights underscore the importance of participatory, community‐led strategies within health communication [65].

### 4.2 Programmatic Focus on Equity

The link between vaccine equity and the dynamics of vaccine confidence, trust and misinformation was highlighted in most study conclusions. Physical access to vaccines, health system failings and the social determinants of health (poverty, discrimination, lack of access to education) were frequently linked to lowered confidence in vaccination [27, 37, 45, 66].

Several sources conclude that public health actors should make vaccine equity central to programming by addressing structural barriers and ensuring that marginalised communities (such as populations with zero‐dose children and those in conflict settings) have access to vaccines [38, 52]. The CCA contends that key to this is a critical engagement with the material realities of the communities that public health responders are trying to reach [64]. Several studies in this scoping review implicitly supported this view, identifying community voices, community‐led interventions and embedding local leaders at the core of programmes. Yet, even partnerships that appear equitable may reproduce power asymmetries [67]. It is acknowledged that in crisis contexts, it is challenging to achieve equitable programmes. In these contexts, with limited or damaged health infrastructure, community health services are critical [38, 39]. In geographically isolated or insecure areas, vaccination programmes are even more precarious [29]. Studies point to investment in mobile clinics, integrated health services and strong partnerships with local organisations and community representatives [49].

Marginalised communities often face additional barriers to vaccination due to poverty, discrimination and lack of access to healthcare and health information [49, 50, 68]. It was recommended that tailored interventions include culturally sensitive outreach and communications campaigns and engagement with trusted information channels and messengers. Although difficult in humanitarian crises, where lifesaving priorities often compete, programmes that address the social determinants of health, such as poverty, education and housing, through interventions that seek to bundle multi‐sectoral ‘packages of care’, may help empower marginalised communities [31, 36]. In crises, addressing immediate threats such as malnutrition, insecurity and lack of shelter may take programmatic and social precedence but can be vehicles in which vaccination can be embedded [29]. Advocacy for sustainable, equitable financing across the humanitarian‐development nexus is essential for building health system equity before, during and after crises [52].

### 4.3 Misinformation as a Dynamic and Contextually Embedded Phenomenon

Much of the literature encourages a framing of the infodemic and misinformation within a wider context. Individual consumption of, or interaction with, misinformation does not occur in isolation, and individuals do not absorb information passively [69, 70, 71]. The interpretation of information occurs through the lens of cultural beliefs, social norms and individual experiences [9, 72].

In the context of crisis, where there has been a rupture of social structures and increasingly asymmetrical power dynamics, (mis)information is also a tool. It helps facilitate sense‐making, often evolving from historical narratives [33, 42]. In the absence of accessible, trustworthy and culturally relevant information, or where official narratives contradict lived experiences, individuals and communities turn to information sources that help them make sense of uncertainty, injustice and the impact of crisis [25, 26, 33].

### 4.4 Strengths and Limitations

The search strategy was designed in consultation with information specialists at the London School of Hygiene and Tropical Medicine Library and incorporated multiple databases and hand searching to identify relevant studies. Additionally, the review included studies employing a variety of research methods. The data were extracted and reviewed by two independent authors, enhancing the validity of findings.

We nevertheless acknowledge some limitations. The choice of search terms and uncertainty in definitions for key themes may have missed some studies, although the hand searching of reference lists aimed to reduce this risk. The search was limited to English language publications, and we concede that some relevant works might have been published in languages other than English.

Non‐peer‐reviewed grey literature was not included, such as those developed by the humanitarian response community, including needs assessments, programme evaluations, toolkits, training modules and guidelines. The exclusion decision was taken given the heterogeneity of grey literature, making it challenging to synthesise findings alongside peer‐reviewed articles consistently. However, synthesising grey literature could provide valuable additional insights into operational strategies and programmatic responses and may be considered in future work.

A large proportion of studies were authored by institutions based in high‐income countries, predominantly the United States and the United Kingdom. Although institutional affiliation often reflects funding and infrastructure, it does not necessarily mean that researchers themselves are not from the countries being studied. Over half of the studies represented co‐authorship with regions from which the research was conducted and institutions in high‐income settings. These partnerships can strengthen contextual relevance and enhance capacity strengthening. However, it highlights ongoing structural inequalities in global health research and the importance of enabling locally led research in humanitarian contexts [73].

### 4.5 Implications for Research and Practice

Despite the valuable insights provided in the review studies, there remain critical knowledge gaps in understanding the interplay of trust, information and vaccine confidence in crisis settings. Future research on misinformation needs to be framed as an exploration of an evolving social phenomenon that exists within context. Within research in humanitarian contexts, there remain gaps in nuance and critical framing of the connection between misinformation and trust and its impact on vaccine confidence. This is likely because the nature of humanitarian crises means research is challenging to carry out and ethical implications are more complex. Several studies emphasised the need for longitudinal studies that track how trust evolves over time and in relation to interventions and changing contexts [6, 28, 35]. Comparative studies across contexts will contribute to building a more robust framework around understanding trust and lend more nuance to explorations of vaccine confidence and misinformation [28, 74].

Expanding the practice of embedding social science research into outbreak and humanitarian response will help build a more complex and detailed picture of vaccinating publics and their perspectives [27, 33]. This is particularly important for clinical vaccine trials in fragile settings. Researchers need to consider cultural nuances, explore power dynamics and notions of fairness with clear commitments to enhance the social value of research and effectively navigate ethical implications [12, 32, 50]. Quantitative data are important to supplement qualitative findings on the relationship between trust‐building efforts and vaccination rates [12]. Additionally, there need to strengthen the evidence base on effective strategies that improved vaccine confidence, particularly through approaches that are participatory, context specific and responsive to community concerns. Overall, sustained, long‐term engagement in building strong health systems and adequate community health structures will be measures that build community trust.

## 5 Conclusion

Trust, information and vaccine confidence are not isolated variables. They are shaped by history, power and social dynamics. Addressing these challenges in humanitarian settings requires sustained, participatory approaches and a research agenda that builds the evidence base for community‐led and equity‐focused programmes.

## Author Contributions

Harriet Dwyer conceptualised and designed the project, collected and analysed the data and wrote the first draft of the article. Shereen Ayub was the project's second reviewer, contributing to independent data extraction and resolving conflicts, as well as critical revisions of the article. Nadine Beckmann, Luisa Enria, and Jennifer Palmer provided overall technical guidance and critical revisions of the article. All authors agreed to the final draft prior to submission.

## Ethics Statement

This review uses data published publicly in peer‐reviewed literature and therefore did not require ethical approval.

## Conflicts of Interest

The authors declare no conflicts of interest.

## Supporting information




**Supplementary file 1**: puh270073‐sup‐0001‐SuppMat.pdf

**Supplementary file 2**: puh270073‐sup‐0002‐SuppMat.pdf

**Supplementary file 2**: puh270073‐sup‐0003‐SuppMat.docx

## Data Availability

Data from the review had been made available as Supporting Information with the submission.

## 1

Table A1.

**TABLE A1:** Search strategy structure.

Concept	(Mis)information	Trust/mistrust	Vaccine confidence
**Synonyms**	Rumo#r*	Hope	Immune#ation trust
	Disinformation	Faith	vaccination acceptance
	‘Fake news’	Reliance	positive vaccine attitude*
	Propagand*	Confidence	Immune#ation belie*
	Infodemic*	Assurance	confidence in vaccination
	Conspirac* or conspire* or conspirator*	Belie*	vaccine hesitancy
	‘False narrative*’	Conviction	Immune#ation reluctan*
	Idiom	Dependence	vaccination hesitan*
	Information cris*	Credence	vaccine skepti*
	Urban legend	Fidelity	doubts about vaccination
		Trustworthiness	Immune#ation uncertainty
		Loyalty	reluctance to vaccinate
		Dependability	vaccine refusal
		Allegiance	vaccine resistance
		Scepticism	
		Distrust	
		Suspicion	
		Disbelief	
		Wariness	
		Doubt	
		Unbelief	
		Cynicism	
		Disillusionment	
		Caution	
		Mistrustfulness	
		Misgiving	
		Uncertainty	
**Subheadings**	propaganda	Trust	Vaccination refusal
	disinformation		Vaccination hesitancy
	gaslighting		anti‐vaccination movement

## Ovid MEDLINE(R) ALL 〈1946 to 21 June 2024〉

1. (misinformation or Rumo#r* or Disinformation or ‘Fake news’ or Propagand* or Infodemic* or Conspirac* or conspire* or conspirator* or ‘False narrative*’ or Idiom or Information cris* or Urban legend).mp. [mp = title, book title, abstract, original title, name of substance word, subject heading word, floating sub‐heading word, keyword heading word, organism supplementary concept word, protocol supplementary concept word, rare disease supplementary concept word, unique identifier, synonyms, population supplementary concept word, anatomy supplementary concept word]: 12358
2. propaganda/ or disinformation/ or gaslighting/: 911
3. 1 or 2: 12367
4. (trust or Hope or Faith or Reliance or Confidence or Assurance or Belie* or Conviction or Dependence or Credence or Fidelity or Trustworthiness or Loyalty or Dependability or Allegiance or Scepticism or Distrust or Suspicion or Disbelief or Wariness or Doubt or Unbelief or Cynicism or Disillusionment or Caution or Mistrustfulness or Misgiving or Uncertainty).mp. [mp = title, book title, abstract, original title, name of substance word, subject heading word, floating sub‐heading word, keyword heading word, organism supplementary concept word, protocol supplementary concept word, rare disease supplementary concept word, unique identifier, synonyms, population supplementary concept word, anatomy supplementary concept word]: 1894493
5. Trust/: 13769
6. 4 or 5: 1894493
7. (vaccine confidence or Immune#ation trust or vaccination acceptance or positive vaccine attitude* or Immune#ation belie* or confidence in vaccination or vaccine hesitancy or Immune#ation reluctan* or vaccination hesitan* or vaccine skepti* or doubts about vaccination or Immune#ation uncertainty or reluctance to vaccinate or vaccine refusal or vaccine resistance).mp. [mp = title, book title, abstract, original title, name of substance word, subject heading word, floating sub‐heading word, keyword heading word, organism supplementary concept word, protocol supplementary concept word, rare disease supplementary concept word, unique identifier, synonyms, population supplementary concept word, anatomy supplementary concept word]: 8477
8. vaccination refusal/ or vaccination hesitancy/: 2011
9. 7 or 8: 8835
10. 3 and 6 and 9531
