# Supplementary material for: Trust, Information and Vaccine Aonfidence in Crisis Settings: A Scoping Review
Source: Public Health Chall. 2025 Jun 26;4(3):e70073. doi: 10.1002/puh2.70073 (PMC12198467; doi:10.1002/puh2.70073)
Supplement: Supplementary file 1 — puh270073‐sup‐0001‐SuppMat.pdf [file PUH2-4-e70073-s003.pdf]

Study ID	Title	First author	Institution country/ countries	Year of pub.	Journal	Aim	Study design	Research country/ countries	Setting	Population	Data collection methods	Key themes
Yahya 2007	**Polio vaccines "no thank you!" barriers to polio eradication in Northern Nigeria**	Maryam Yahya	Nigeria	2007	African Affairs	To analyze the causes of the polio vaccination boycott in northern Nigeria and its effects on global polio eradication efforts.	Qualitative research	Nigeria	Epidemic (outbreak in fragile context e.g. Ebola, cholera)	Communities in Northern Nigeria, particularly Muslim communities influenced by political and religious leaders.	Interviews; Focus groups; Observation; Other: Desk research	(mis)information (e.g., unverified information, rumours, disinformation); vaccine sentiments (confidence, hesitancy etc.); trust (e.g., reliance on information sources, social trust dynamics); humanitarian response
Vinck 2019	**Institutional trust and misinformation in the response to the 2018-19 Ebola outbreak in North Kivu, DR Congo: a population-based survey**	Patrick Vinck	DR Congo, Democratic Republic of the Congo, UK, USA	2019	Lancet Infectious Disease	To investigate the role of institutional trust and misinformation in influencing individual preventive behaviors during the 2018â€"19 Ebola virus disease (EVD) outbreak in North Kivu, Democratic Republic of Congo (DRC).	Cross sectional study	North Kivu, Democratic Republic of Congo	Epidemic (outbreak in fragile context e.g. Ebola, cholera)	Adults residing in the cities of Beni and Butembo in North Kivu, DRC	Survey	(mis)information (e.g., unverified information, rumours, disinformation); vaccine sentiments (confidence, hesitancy etc.); trust (e.g., reliance on information sources, social trust dynamics); humanitarian response ; Other: Preventive behaviours
Obadare 2005	**A crisis of trust: history, politics, religion and the polio controversy in Northern Nigeria**	Ebenezer Obadare	UK	2005	Patterns of Prejudice	To explore the complex dynamics of trust, politics, religion, and health interventions during the polio vaccination controversy in Northern Nigeria.	Qualitative research	Nigeria	Epidemic (outbreak in fragile context e.g. Ebola, cholera)	Communities in Northern Nigeria, especially Muslim populations resistant to polio vaccination.	Document analysis	(mis)information (e.g., unverified information, rumours, disinformation); vaccine sentiments (confidence, hesitancy etc.); trust (e.g., reliance on information sources, social trust dynamics)
Ismail 2022	**Strengthening vaccination delivery system resilience in the context of protracted humanitarian crisis: a realist-informed systematic review**	Sharif A. Ismail	Lebanon, Singapore, Switzerland, UK	2022	BMC Health Services Research	To critically evaluate the evidence on the effectiveness of system-level interventions for improving vaccination coverage in protracted humanitarian crises, focusing on how they work and for whom, to better inform preparedness and response for future crises.	Systematic review	Global analysis	Global analysis	Populations in humanitarian crises, including refugees, internally displaced persons (IDPs), and host communities, primarily focusing on children aged 0-5 years.	Document analysis ; Other: Systematic review of peer-reviewed and grey literature, with keyword-structured searches performed in multiple databases and grey literature sources. Findings were narratively synthesized.	vaccine sentiments (confidence, hesitancy etc.); trust (e.g., reliance on information sources, social trust dynamics); humanitarian response
Folayan 2016	**Ebola vaccine development plan: ethics, concerns and proposed measures**	Morenike Oluwatoyin Folayan	Australia, Nigeria, USA	2016	BMC Medical Ethics	To discuss ethical concerns and suggest measures for addressing these concerns in the context of emergency Ebola vaccine development which specifically targets healthcare workers.	Text, commentary, letter and opinion	Not specific to one country; it discusses issues relevant to the global context, with a focus on West Africa (e.g., Guinea, Sierra	Epidemic (outbreak in fragile context e.g. Ebola, cholera)	Healthcare workers, and other populations at high risk of Ebola infection.	Other: Analysis and discussion based on literature review and ethical frameworks.	(mis)information (e.g., unverified information, rumours, disinformation); vaccine sentiments (confidence, hesitancy etc.); trust (e.g., reliance on information sources, social trust dynamics); humanitarian response
Enria 2016	**Power, fairness and trust: understanding and engaging with vaccine trial participants and communities in the setting up the EBOVAC-Salone vaccine trial in Sierra Leone**	Luisa Enria	Sierra Leone, Tanzania, UK	2016	BMC Public Health	To discuss the establishment of the EBOVAC-Salone clinical trial of an Ebola vaccine candidate in Kambia District, Sierra Leone during the Ebola epidemic, and to analyze the role of social science research in ensuring effective community engagement strategies by incorporating lessons from the socio-political context and the recent Ebola outbreak.	Qualitative research	Sierra Leone	Epidemic (outbreak in fragile context e.g. Ebola, cholera)	Communities in Kambia District, Northern Sierra Leone, including individuals who participated in the EBOVAC-Salone vaccine trial.	Interviews; Focus groups; Other: Ethnographic observation, key stakeholder interviews, exit interviews with trial participants	(mis)information (e.g., unverified information, rumours, disinformation); vaccine sentiments (confidence, hesitancy etc.); trust (e.g., reliance on information sources, social trust dynamics); Other: Power dynamics within communities
Ali 2019	**Polio vaccination controversy in Pakistan**	Mohammad Ali	Pakistan	2019	The Lancet	To address and comment on the barriers preventing polio vaccination and eradication in Pakistan, linked to religious extremism and global political interests, and to discuss the measures taken by the Pakistani government to manage these issues.	Text, commentary, letter and opinion	Pakistan	Epidemic (outbreak in fragile context e.g. Ebola, cholera)	The general population of Pakistan, but particularly focused on communities in Khyber Pakhtunkhwa province impacted by misinformation and resistance to polio vaccination.	Other: Not applicable (commentary/opinion piece)	(mis)information (e.g., unverified information, rumours, disinformation); vaccine sentiments (confidence, hesitancy etc.); trust (e.g., reliance on information sources, social trust dynamics); humanitarian response
Alenichev 2020	**Conceptions within misconceptions: Pluralisms in an Ebola vaccine trial in West Africa**	Arsenii Alenichev	Belgium, Netherlands, Spain	2020	Global Public Health	To explore participantsâ€™ understanding of trial procedures for an experimental vaccine against Ebola virus disease (EVD) in a West African context, focusing on the plurality of knowledge and misconceptions surrounding the vaccine trial.	Qualitative research	West Africa (specifically Liberia)	Epidemic (outbreak in fragile context e.g. Ebola, cholera)	Participants of the Ebola vaccine trial in Liberia, including community members who were part of the trial.	Interviews; Focus groups; Document analysis ; Observation	(mis)information (e.g., unverified information, rumours, disinformation); vaccine sentiments (confidence, hesitancy etc.); trust (e.g., reliance on information sources, social trust dynamics); humanitarian response
ZoaibHabib 2022	**Double trouble: COVID-19 vaccine misinformation amidst conflict in Ukraine**	Tharwani Zoaib Habib	Afghanistan, Pakistan, Ukraine, United Arab Emirates	2022	Annals of Medicine and Surgery	To highlight the severe impact of vaccine misinformation on COVID-19 vaccination efforts during the ongoing conflict in Ukraine. The article seeks to emphasize the compounded challenges posed by misinformation and the conflict, and to propose strategies for improving vaccine acceptance and public health responses under such difficult circumstancesâ€‹.	Text, commentary, letter and opinion	Ukraine	Covid-19 within a fragile context	Ukrainian communities impacted by the conflict	Document analysis ; Other: Review of existing literature and analysis of the impact of misinformation on COVID-19 vaccine acceptance	(mis)information (e.g., unverified information, rumours, disinformation); infodemic; vaccine sentiments (confidence, hesitancy etc.); trust (e.g., reliance on information sources, social trust dynamics); humanitarian response
Steinke 2021	**Whose Health Matters: Trust and Mistrust in Humanitarian Crisis and Global Health Interventions**	Andrea Steinke	Germany	2021	Handbook of Global Health	To explore the dynamics of trust and mistrust in humanitarian health interventions by analysing three case studies: the cholera epidemic in post-earthquake Haiti, the Ebola outbreaks in West Africa and the Democratic Republic of Congo, and the COVID-19 pandemic.	Qualitative research	Haiti, West Africa, Democratic Republic of Congo, and global	Epidemic (outbreak in fragile context e.g. Ebola, cholera)	Communities impacted by humanitarian crises, including those affected by cholera, Ebola, and COVID-19	Interviews; Document analysis ; Other: Ethnographic research	(mis)information (e.g., unverified information, rumours, disinformation); trust (e.g., reliance on information sources, social trust dynamics); humanitarian response ; Other: Post-colonial and neo-colonial influences

Rzymski 2022	**Vaccination of Ukrainian Refugees: Need for Urgent Action**	Piotr Rzymski	Poland, Ukraine	2022	Clinical Infectious Diseases	To discuss the potential epidemiological risks associated with the war-induced influx of Ukrainian refugees and highlight the need for their swift management through institutional support, educational campaigns, counteracting antiscience misinformation, and pursuing vaccinations.	Text, commentary, letter and opinion	Ukraine	Conflict	Ukrainian refugees, including children and high-risk individuals (elderly, obese, those with comorbidities)	Document analysis	(mis)information (e.g., unverified information, rumours, disinformation); vaccine sentiments (confidence, hesitancy etc.); trust (e.g., reliance on information sources, social trust dynamics); humanitarian response
Leyland 2023	**Misinformation in Humanitarian Programmes**	Jake Leyland	Belgium, India	2023	Journal of Humanitarian Affairs	To better understand and respond to health misinformation in humanitarian settings through the deployment and evaluation of the 'MSF Listen' platform during the COVID-19 pandemic.	Case report	Tajikistan, Afghanistan, Somalia, Latin America, Nigeria, and Haiti.	Global analysis	MSF teams	Survey; Interviews; Document analysis	(mis)information (e.g., unverified information, rumours, disinformation); trust (e.g., reliance on information sources, social trust dynamics); humanitarian response
Gidado 2024	**Knowledge, risk perception and uptake of COVID-19 vaccination among internally displaced persons in complex humanitarian emergency setting, Northeast Nigeria**	Saheed Gidado	Nigeria	2024	BMC Public Health	To assess COVID-19 knowledge, risk perception, and vaccination uptake among internally displaced persons (IDPs) in Northeast Nigeria, and to investigate the association between risk perception and COVID-19 preventive measures.	Cross sectional study	Nigeria	Covid-19 within a fragile context	Internally Displaced Persons (IDPs) residing in camps in Borno, Adamawa, and Yobe States	Survey; Interviews; Other: Stratified random sampling	(mis)information (e.g., unverified information, rumours, disinformation); vaccine sentiments (confidence, hesitancy etc.); trust (e.g., reliance on information sources, social trust dynamics); humanitarian response
Dhaliwal 2024	**Introduction of the pneumococcal conjugate vaccine in humanitarian and fragile contexts: Perspectives from stakeholders in four African countries**	Baldeep K. Dhaliwal	Chad, Guinea, Somalia, South Sudan, USA	2024	Human Vaccines & Immunotherapeutics	To understand the factors that affect the introduction and implementation of the pneumococcal conjugate vaccine (PCV) in Gavi-eligible countries with high levels of childhood pneumonia deaths, including Chad, Guinea, Somalia, and South Sudan.	Qualitative research	Chad, Guinea, Somalia, and South Sudan	Conflict	Policymakers and leaders of implementation efforts, including immunization program managers, Ministry of Health officials, and in-country implementing organizations.	Interviews	trust (e.g., reliance on information sources, social trust dynamics); Other: Population-level vulnerabilities to pneumonia, disease burden, policy processes, and vaccine introduction optimization
Aarslew 2023	**Despite misinformation, low trust, and conflict in Somalia, high demand for vaccines and a negative endorsement effect of non-state authorities**	Laurits F. Aarslew	Denmark, UK	2023	Scientific Reports	To better understand the levels and drivers of COVID-19 vaccine receptivity in conflict regions, specifically South Central Somalia.	Other: Longitudinal study	Somalia	Covid-19 within a fragile context	Communities impacted by conflict in South Central Somalia	Survey	(mis)information (e.g., unverified information, rumours, disinformation); vaccine sentiments (confidence, hesitancy etc.); trust (e.g., reliance on information sources, social trust dynamics)
Gostin 2020	**The public health crisis of underimmunisation: a global plan of action**	Lawrence O Gostin	Poland, UK, USA	2020	Lancet Infectious Diseases	To address the global public health crisis of underimmunisation, identify its causes, and provide a global plan of action to increase vaccination rates and reduce vaccine-preventable diseases.	Text, commentary, letter and opinion	The study covers a global analysis, with examples from countries like the United States, Nigeria, and France.	Global analysis	General global populations with a focus on children, particularly in low- and middle-income countries (LMICs), as well as populations affected by conflict and humanitarian crises.	Document analysis	(mis)information (e.g., unverified information, rumours, disinformation); vaccine sentiments (confidence, hesitancy etc.); trust (e.g., reliance on information sources, social trust dynamics)
Truong 2021	**What factors promote vaccine hesitancy or acceptance during pandemics? A systematic review and thematic analysis**	Judy Truong	Canada, USA	2022	Health Promotion International	To examine the factors that promote vaccine hesitancy or acceptance during pandemics, major epidemics, and global outbreaks through a systematic review and thematic analysis of studies related to Influenza A/H1N1 and Ebola Virus Disease.	Systematic review	Includes studies from various countries, including European countries, Canada, the USA, African countries (Guinea, Uganda, Sierra Leone), Australia, and Asian countries (China,	Global analysis	The review includes studies from various populations across multiple countries affected by Influenza A/H1N1 and Ebola, including European, North American, African, and Asian countries. The populations discussed are general public respondents, healthcare workers, and at-risk groups.	Other: Systematic review of studies followed by qualitative thematic analysis	(mis)information (e.g., unverified information, rumours, disinformation); vaccine sentiments (confidence, hesitancy etc.); trust (e.g., reliance on information sources, social trust dynamics)
Douedari 2023	**COVID-19 is just another way to die...: a qualitative longitudinal study of frontline COVID-19 response governance across Syria**	Yazan Douedari	Singapore, Syria, UK	2023	BMJ Global Health	To explore perspectives of COVID-19 response governance among frontline healthcare providers over time and across major areas of control in Syria.	Qualitative research	Syria	Covid-19 within a fragile context	Frontline healthcare providers	Interviews; Other: Integrative thematic analysis.	(mis)information (e.g., unverified information, rumours, disinformation); vaccine sentiments (confidence, hesitancy etc.); trust (e.g., reliance on information sources, social trust dynamics); humanitarian response
Vanderslott 2022	**Attributing public ignorance in vaccination narratives**	Samantha Vanderslott	Sierra Leone, UK	2022	Social Science & Medicine	To explore the framing of an 'ignorant public' in vaccination narratives in public discourse and how this oversimplifies complex socio-political factors; obscuring mistrust and deeper concerns about vaccine deployment.	Qualitative research	Sierra Leone, Uganda, India	Other: Vaccination campaigns related to Ebola, measles-rubella, and African Swine Fever (ASF)	The populations examined include rural and border communities in Sierra Leone, subsistence farmers in Uganda, and minority Muslim groups in India.	Survey; Interviews; Document analysis ; Observation	(mis)information (e.g., unverified information, rumours, disinformation); vaccine sentiments (confidence, hesitancy etc.); trust (e.g., reliance on information sources, social trust dynamics)
Samadi 2023	**Attitude and acceptance toward COVID-19 vaccines among Kabul cityâ€™s residents: A cross sectional study**	Assadullah Samadi	Afghanistan	2023	Turkish Journal of Public Health	To assess the attitude and perception of Kabul city residents toward COVID-19 vaccine hesitancy and acceptance, and to identify the factors influencing these attitudes.	Cross sectional study	Afghanistan	Covid-19 within a fragile context	Residents of Kabul city, Afghanistan	Survey	(mis)information (e.g., unverified information, rumours, disinformation); vaccine sentiments (confidence, hesitancy etc.); trust (e.g., reliance on information sources, social trust dynamics); humanitarian response

Lasco 2020	**Medical populism and immunisation programmes: Illustrative examples and consequences for public health**	Gideon Lasco	Philippines, UK, USA	2020	Global Public Health	The paper aims to explore the dynamics of how vaccine hesitancy and immunisation programs are politicized, exacerbating negative attitudes toward vaccines and contributing to retrogressive policies.	Text, commentary, letter and opinion	Nigeria, Italy, Ukraine, and the Philippines.	Epidemic (outbreak in fragile context e.g. Ebola, cholera)	Nigeria: Communities in northern Nigeria, particularly during the polio vaccine boycott of 2003.<br><br>Italy: The general population influenced by anti-vaccine political rhetoric during the 2015 elections.<br><br>Ukraine: The public and health authorities affected by the measles-rubella (MR) vaccine scare in 2008.<br><br>The Philippines: Communities affected by the Dengvaxia vaccine controversy in 2017.	Document analysis	vaccine sentiments (confidence, hesitancy etc.); trust (e.g., reliance on information sources, social trust dynamics)
Ittefaq 2021	**Polio vaccine misinformation on social media: turning point in the fight against polio eradication in Pakistan**	Muhammad Ittefaq	Pakistan, USA	2021	Human Vaccines & Immunotherapeutics	To explore the impact of polio vaccine misinformation on social media and its consequences on polio eradication efforts in Pakistan.	Text, commentary, letter and opinion	Pakistan	Epidemic (outbreak in fragile context e.g. Ebola, cholera)	Communities in polio endemic areas of Pakistan	Other: Analysis and discussion based on literature review and observation of social media trends.	(mis)information (e.g., unverified information, rumours, disinformation); vaccine sentiments (confidence, hesitancy etc.); trust (e.g., reliance on information sources, social trust dynamics); humanitarian response
Haq 2024	**Building confidence in the COVID-19 vaccine in a polio-endemic country: strategic communication lessons from Pakistan**	Zaeem Ul Haq	Pakistan, USA	2024	BMJ Global Health	To explore the strategic communication approaches used in Pakistan to build confidence in the COVID-19 vaccine, particularly in the context of a polio-endemic country with a history of vaccine hesitancy.	Cross sectional study	Pakistan	Covid-19 within a fragile context	General population of Pakistan, with a focus on areas with historical vaccine hesitancy	Document analysis ; Secondary data; Other: Analysis of communication strategies and their effectiveness based on historical data and outcomes from the COVID-19 vaccination campaign in Pakistan	(mis)information (e.g., unverified information, rumours, disinformation); infodemic; vaccine sentiments (confidence, hesitancy etc.); trust (e.g., reliance on information sources, social trust dynamics); humanitarian response ; Other: Strategic communications
Abdullahi 2020	**Factors contributing to the uptake of childhood vaccination in Galkayo District, Puntland, Somalia**	Mohamed Farah Abdullahi	Australia, Somalia, Sweden	2020	Global Health Action	To investigate factors associated with childhood vaccination uptake from the perspectives of both communities and health care workers in Galkayo District, Somalia.	Qualitative research	Somalia	Conflict	Communities impacted by humanitarian crisis, specifically children under five years old in Galkayo District, Puntland, Somalia.	Survey; Interviews; Focus groups	(mis)information (e.g., unverified information, rumours, disinformation); vaccine sentiments (confidence, hesitancy etc.); trust (e.g., reliance on information sources, social trust dynamics); humanitarian response
James 2023	**Protection, health seeking, or a laissez-passer: Participants? decision-making in an EVD vaccine trial in the eastern Democratic Republic of the Congo**	Myfanwy James	DRC, France, Tanzania, United Kingdom	2023	Social Science & Medicine	To analyse the motivations of participants who volunteered for the DRC-EB-001 Ebola vaccine trial in North Kivu, Democratic Republic of the Congo (DRC), during the 10th Ebola virus epidemic.	Qualitative research	Democratic Republic of the Congo (DRC)	Epidemic (outbreak in fragile context e.g. Ebola, cholera)	Participants of the DRC-EB-001 EVD vaccine trial in Goma, North Kivu, including local communities with a history of conflict and exposure to epidemics.	Interviews; Focus groups; Observation	(mis)information (e.g., unverified information, rumours, disinformation); vaccine sentiments (confidence, hesitancy etc.); trust (e.g., reliance on information sources, social trust dynamics); humanitarian response
Heyerdahl 2023	**Parallel vaccine discourses in Guinea: 'grounding' social listening for a non-hegemonic global health**	Leonardo W Heyerdahl	Belgium, France, Guinea, UK	2023	Critical Public Health	The study aims to develop a rich understanding of vaccine sentiments and decisions among Guinean healthcare workers by incorporating socially-embedded knowledge and taking a non-normative stance that focuses on openly discussing vaccine sentiments rather than classifying claims as accurate or not.	Qualitative research	Guinea	Covid-19 within a fragile context	Health workers, social workers, general population	Interviews; Other: 'Grounded social listening'	(mis)information (e.g., unverified information, rumours, disinformation); vaccine sentiments (confidence, hesitancy etc.); trust (e.g., reliance on information sources, social trust dynamics); humanitarian response ; Other: Politicization of vaccine rollout
Alhaffar 2022	**"<i>They cannot afford to feed their children and the advice is to stay home</i>. <i>How</i>??": A qualitative study of community experiences of COVID-19 response efforts across Syria**	Mervat Alhaffar	Singapore, Syria, United Kingdom	2022	PLOS ONE	To examine public perceptions about the effects of the COVID-19 pandemic and health authority responses in Syriaâ€™s three main areas of control.	Qualitative research	Syria	Covid-19 within a fragile context	Syrian nationals living in Syria during the COVID-19 pandemic, aged 18 years or over	Interviews	(mis)information (e.g., unverified information, rumours, disinformation); vaccine sentiments (confidence, hesitancy etc.); trust (e.g., reliance on information sources, social trust dynamics)
Wonodi 2022	**Conspiracy theories and misinformation about COVID-19 in Nigeria: Implications for vaccine demand generation communications**	Chizoba Wonodi	Nigeria, United States	2022	Vaccine	To systematically analyse the prevalence and nature of conspiracy theories and misinformation about COVID-19 in Nigeria, and assess how these narratives influence vaccine hesitancy. The goal is to provide insights to guide effective communication strategies to improve vaccine acceptance.	Qualitative research	Nigeria	Covid-19 within a fragile context	Community members, healthcare workers, program managers, and religious leaders.	Interviews; Focus groups	(mis)information (e.g., unverified information, rumours, disinformation); infodemic; vaccine sentiments (confidence, hesitancy etc.); trust (e.g., reliance on information sources, social trust dynamics)
Wardak 2021	**COVID-19 vaccination efforts: Is Afghanistan prepared?**	Mohammad Faisal Wardak	Afghanistan, United Kingdom	2021	The American Journal of Tropical Medicine and Hygiene	The study aimed to analyze Afghanistanâ€™s preparedness for COVID-19 vaccination efforts and the challenges ahead, along with recommendations on overcoming these challenges.	Text, commentary, letter and opinion	Afghanistan	Covid-19 within a fragile context	All Afghans, with a focus on high-risk groups such as healthcare workers, security personnel, people with comorbidities, and individuals in remote or conflict-affected areas.	Observation; Other: Analysis based on existing literature and studies	(mis)information (e.g., unverified information, rumours, disinformation); infodemic; vaccine sentiments (confidence, hesitancy etc.); trust (e.g., reliance on information sources, social trust dynamics); humanitarian response
Shibani 2021	**COVID-19 vaccination acceptance among Syrian population: a nationwide cross-sectional study**	Mosa Shibani	France, Russia, Qatar, Syria, USA	2021	BMC Public Health	To assess the willingness of the Syrian population to receive the COVID-19 vaccine and identify the factors influencing their decision-making process.	Cross sectional study	Syria	Covid-19 within a fragile context	A nationally representative sample of Syrian	Survey	(mis)information (e.g., unverified information, rumours, disinformation); infodemic; vaccine sentiments (confidence, hesitancy etc.); trust (e.g., reliance on information sources, social trust dynamics)

Ghinai 2013	**Listening to the rumours: What the northern Nigeria polio vaccine boycott can tell us ten years on**	Isaac Ghinai	Nigeria, UK	2013	Global Public Health	The article aims to analyse the reasons behind the polio vaccine boycott in northern Nigeria in 2003-2004, focusing on local political, religious, and social factors that fuelled vaccine rumours and resistance.	Qualitative research	Nigeria	Epidemic (outbreak in fragile context e.g. Ebola, cholera)	Communities in northern Nigeria, particularly those in Kano State, including political and religious leaders, healthcare professionals, and the general population	Interviews; Document analysis	(mis)information (e.g., unverified information, rumours, disinformation); vaccine sentiments (confidence, hesitancy etc.); trust (e.g., reliance on information sources, social trust dynamics); humanitarian response ; Other: Religious and ethnic influences on health behaviors, and political dynamics and their impact on public health interventions
Etienne-Mesubi 2024	**Factors that influence COVID-19 Vaccine Uptake and Hesitancy Among a Population in the West Department of Haiti: Implications for Enhancing Effectiveness of Immunization Programs**	Martine Etienne-Mesubi	United States	2024	medRxiv (preprint)	To evaluate population characteristics related to vaccine uptake and hesitancy in the West Department of Haiti to inform future interventions and strategies for improving COVID-19 vaccine uptake and safeguarding against future global health security threats.	Cross sectional study	Haiti	Covid-19 within a fragile context	Adults residing in peri-urban communes of the West Department of Haiti	Survey; Focus groups	(mis)information (e.g., unverified information, rumours, disinformation); vaccine sentiments (confidence, hesitancy etc.); trust (e.g., reliance on information sources, social trust dynamics); Other: Influence of religious leaders and social norms on vaccination decisions
Underwood 2023	**Shaping global vaccine acceptance with localized knowledge: a report from the inaugural VARN2022 conference**	Talya Underwood	Australia, Canada, Kenya, Pakistan, UK, USA, Uganda	2023	BMC Proceedings	To summarize the insights and discussions from the inaugural Vaccination Acceptance Research Network (VARN2022) conference, which aimed to address global vaccine acceptance through localized knowledge and context-specific strategies.	Other: Meeting report	The report includes insights and data from various countries across the globe, with a particular focus on low- and middle-income countries	Global analysis	The conference covered diverse populations, including marginalized communities, Indigenous populations, and people in LMICs, with a focus on their perspectives on vaccine acceptance.	Other: The report is based on presentations, discussions, and thematic analyses conducted during the VARN2022 conference. It includes qualitative summaries of insights and evidence presented by various global experts.	(mis)information (e.g., unverified information, rumours, disinformation); infodemic; vaccine sentiments (confidence, hesitancy etc.); trust (e.g., reliance on information sources, social trust dynamics)
Tabong 2022	**Preparation for COVID-19 vaccines rollout: Interventions to increase trust, acceptability, and uptake in West African countries**	Philip Teg-Nefaah Tabong	Ghana	2022	International Journal of Health Planning and Management	The paper aims to propose interventions to increase trust, acceptability, and uptake of COVID-19 vaccines in West African countries, drawing lessons from previous vaccination exercises for emerging and re-emerging infectious diseases.	Text, commentary, letter and opinion	West African countries.	Covid-19 within a fragile context	The population focus is the general public in West African countries, particularly rural communities, and hard-to-reach populations.	Document analysis	(mis)information (e.g., unverified information, rumours, disinformation); vaccine sentiments (confidence, hesitancy etc.); trust (e.g., reliance on information sources, social trust dynamics); humanitarian response
Nyalundja 2024	**COVID-19 Vaccine Hesitancy and Associated Oral Cholera Vaccine Hesitancy in a Cholera-Endemic Country: A Community-Based Cross-Sectional Study in the Democratic Republic of Congo**	Arsene Daniel Nyalundja	Democratic Republic of the Congo, South Africa, USA	2024	Vaccines	To assess the impact of COVID-19 vaccine hesitancy and its drivers on oral cholera vaccine (OCV) hesitancy in a cholera-endemic region of the Democratic Republic of Congo (DRC) and to understand the factors contributing to vaccine hesitancy in this context.	Cross sectional study	Democratic Republic of Congo (DRC)	Epidemic (outbreak in fragile context e.g. Ebola, cholera)	Adults aged 18 and above in Bukavu, South Kivu, DRC	Survey	(mis)information (e.g., unverified information, rumours, disinformation); infodemic; vaccine sentiments (confidence, hesitancy etc.); trust (e.g., reliance on information sources, social trust dynamics); humanitarian response
Mohamad 2021	**Factors associated with the intention of Syrian adult population to accept COVID19 vaccination: a cross-sectional study**	Okbah Mohamad	Syria	2021	BMC Public Health	To estimate the proportion of the Syrian adult population intending to be vaccinated against COVID-19 and to assess the demographic and attitudinal factors associated with vaccination intentions in order to develop suitable interventions.	Cross sectional study	Syria	Covid-19 within a fragile context	Syrian adults across various provinces, recruited through social media platforms	Survey	(mis)information (e.g., unverified information, rumours, disinformation); vaccine sentiments (confidence, hesitancy etc.); trust (e.g., reliance on information sources, social trust dynamics)
Hopkins 2024	**The second annual Vaccination Acceptance Research Network Conference (VARN2023): Shifting the immunization narrative to center equity and community expertise**	Kathryn L Hopkins	Australia, Canada, Pakistan, Switzerland, UK, USA	2024	Vaccine	To synthesize and share evidence-based insights from the second annual Vaccination Acceptance Research Network Conference (VARN2023) focused on improving vaccine acceptance, demand, and delivery through equity-centered, community-led approaches.	Text, commentary, letter and opinion	Global (including insights from over 40 countries)	Global analysis	Global stakeholders including researchers, practitioners, and community members from low-and middle-income countries (LMICs) as well as representatives from marginalized communities	Document analysis ; Other: Synthesis of oral and poster presentations, topics of discussion from the conference, and evidence-based insights	(mis)information (e.g., unverified information, rumours, disinformation); vaccine sentiments (confidence, hesitancy etc.); trust (e.g., reliance on information sources, social trust dynamics); Other: Social and behavioral science approaches to immunization, vaccine demand and service intergation,
Enria 2024	**Political dimensions of misinformation, trust, and vaccine confidence in a digital age**	Luisa Enria	UK, Sierra Leone	2024	The BMJ	To explore how political dimensions influence vaccine confidence, mistrust, and the spread of misinformation in the context of a digital age.	Text, commentary, letter and opinion	Global	Global analysis	The populations discussed in the article range from marginalized groups in Sierra Leone to Black American communities in the U.S., as well as minority groups such as ultra-Orthodox Jewish communities and political groups in Europe. The article also addresses global patterns of vaccine confidence and hesitancy, especially in online spaces.	Document analysis ; Other: Literature review, case study examples	(mis)information (e.g., unverified information, rumours, disinformation); infodemic; vaccine sentiments (confidence, hesitancy etc.); trust (e.g., reliance on information sources, social trust dynamics); humanitarian response ; Other: Political influences on health outcomes
Enria 2021	**Bringing the social into vaccination research: Community-led ethnography and trust-building in immunization programs in Sierra Leone**	Luisa Enria	United Kingdom, Sierra Leone	2021	PLOS ONE	To explore how community-led ethnographic research can enhance trust and improve vaccine confidence in immunization programs in Sierra Leone	Qualitative research	Sierra Leone	Epidemic (outbreak in fragile context e.g. Ebola, cholera)	Communities in five border towns or villages in Kambia District, Sierra Leone	Interviews; Focus groups; Other: Participant observation, participatory power mapping, rumour tracking	(mis)information (e.g., unverified information, rumours, disinformation); vaccine sentiments (confidence, hesitancy etc.); trust (e.g., reliance on information sources, social trust dynamics); humanitarian response
Chery 2023	**COVID-19 vaccine acceptance in three rural communes in Haiti: A cross-sectional study**	Maurice J. Chery	Haiti, Lesotho, Liberia, Mexico, Peru, Rwanda, USA	2023	Human Vaccines & Immunotherapeutics	To assess COVID-19 vaccine acceptance and hesitancy within three rural communes in Haiti.	Cross sectional study	Haiti	Covid-19 within a fragile context	The general population of three rural communes in Haiti: Hinche, Mirebalais, and Saint-Marc	Survey	(mis)information (e.g., unverified information, rumours, disinformation); vaccine sentiments (confidence, hesitancy etc.); trust (e.g., reliance on information sources, social trust dynamics)
Bitar 2021	**Misinformation, perceptions towards COVID-19 and willingness to be vaccinated: A population-based survey in Yemen**	Ahmad Naoras Bitar	Jordan, Malaysia, United Arab Emirates, Yemen	2021	PLOS ONE	To investigate the prevalence of COVID-19 misinformation among the Yemeni population and its association with vaccine acceptance and perceptions.	Cross sectional study	Yemen	Covid-19 within a fragile context	Yemeni general population	Survey	(mis)information (e.g., unverified information, rumours, disinformation); infodemic; vaccine sentiments (confidence, hesitancy etc.); trust (e.g., reliance on information sources, social trust dynamics); humanitarian response
