# Supplementary material for: Trust, Information and Vaccine Aonfidence in Crisis Settings: A Scoping Review
Source: Public Health Chall. 2025 Jun 26;4(3):e70073. doi: 10.1002/puh2.70073 (PMC12198467; doi:10.1002/puh2.70073)
Supplement: Supplementary file 2 — puh270073‐sup‐0002‐SuppMat.pdf [file PUH2-4-e70073-s001.pdf]

Data Extraction Template (pulled from Covidence)

Study Identification

- **Title:**
- **First Author**:
- **Year of Publication**:
- **Title**:
- **Journal**:
- **DOI**:

Study Characteristics

- **Aim:**
- **Study Design**: (e.g., qualitative, quantitative, mixed methods)
- **Country**:
- **Setting**: (e.g., humanitarian crisis, conflict, outbreak in fragile context)
- **Population**: (e.g., communities impacted by humanitarian crises)
- **Sample Size**:

Methods

- **Data Collection Methods**:
  - Survey
  - Interviews
  - Focus groups
  - Document analysis
  - Experiment
  - Observation
  - Secondary data
  - Other

Key Themes

- **(Mis)information**: (e.g., unverified information, rumours, disinformation)
- **Infodemic**: (e.g., spread of misinformation in public health)
- **Vaccine Confidence**: (e.g., public trust in vaccines, hesitancy)
- **Trust**: (e.g., reliance on information sources, social trust dynamics)

Discussion and results

- **Summary of Findings**: (detailed summary of the main findings)
- **Direct Quotes**: (relevant quotes from participants, if applicable)

Conclusions

- **Authors' Conclusions**:
- **Implications for Practice**: (e.g., recommendations for public health practice)
- **Implications for Research**: (e.g., identified gaps, future research directions)

Additional Information

- **Funding Source**: (if mentioned)
- **Conflicts of Interest**: (if mentioned)
- **Notes**: (any additional notes or comments)
